# Advances of Caspase-11 for veterinary respiratory infections

**DOI:** 10.3389/fvets.2026.1891521

**Published:** 2026-07-17

**Authors:** Yang Su, Kehamo Abi, Qibing Gu, Falong Yang, Guangfu Zhao

**Affiliations:** College of Animal & Veterinary Sciences, Southwest Minzu University, Chengdu, China

**Keywords:** caspase-11, innate immunity, livestock, non-canonical inflammasome, respiratory pathogens

## Abstract

Respiratory diseases represent a major health burden in global livestock production and are driven by bacterial and viral pathogens that trigger severe inflammatory responses and substantial economic losses. Caspase-11 serves as a core component of the non-canonical inflammasome, functioning as an intracellular lipopolysaccharide (LPS) sensor that mediates pyroptosis and inflammatory cytokine maturation. Although extensively studied in human and murine systems, the role of Caspase-11 in veterinary respiratory infections remains poorly characterized. This review synthesizes current knowledge on the structural and regulatory features of the Caspase-11 non-canonical inflammasome, with an emphasis on its involvement in infections caused by major veterinary pathogens, such as *Pasteurella multocida* and *Streptococcus suis*. We further evaluated emerging Caspase-11-targeted therapeutic strategies, including anti-inflammatory agents, vaccine adjuvants, and gene-editing approaches, while critically assessing their translational potential in livestock species. By delineating key knowledge gaps, this review highlights the imperative for species-specific functional studies to inform the development of effective interventions against veterinary respiratory diseases.

## 1 Introduction

Respiratory diseases remain among the most significant health burdens in livestock production worldwide and are caused predominantly by bacterial and viral pathogens that impair host defenses, trigger severe inflammatory responses, and exacerbate tissue damage (1, 2). These pathological changes result in immune dysregulation and metabolic disturbances, collectively impairing production performance and increasing mortality, particularly in young animals. Furthermore, they increase disease prevention and treatment costs, increase pathogen transmission risks, and increase culling rates, ultimately imposing substantial economic burdens on the livestock industry (3–7).

Inflammasomes are multiprotein signaling platforms that are assembled around cytoplasmic pattern recognition receptors, and serve as critical hubs that mediate innate immune inflammatory signal transduction (8). Caspase-11 (official murine gene symbol Caspase-4) is a core component of the non-canonical inflammasome, plays a pivotal role in host defense against gram-negative bacteria. In this review, we retain the term “Caspase-11” to maintain consistency with the majority of cited literature on non-canonical inflammasome activation. It is directly activated by intracellular lipopolysaccharide independent of the TLR4-MyD88 signaling pathway, although its protein expression is regulated by the TLR4-NF-κB axis. Upon activation, Caspase-11 cleaves Gasdermin D (GSDMD), triggering pyroptosis and facilitating the release of IL-1β and IL-18 (9–17).

While the role of the Caspase-11 non-canonical inflammasome in the pathogenesis of human and murine respiratory diseases is well known (16, 18–20), its involvement in veterinary respiratory pathogen infections remains poorly understood. Different pathogens employ distinct infection strategies, and their interactions with Caspase-11 likely exhibit considerable heterogeneity. Moreover, species-specific differences in Caspase-11 homologs across livestock further complicate direct extrapolation from rodent models.

This review systematically summarizes the activation and regulatory mechanisms of the Caspase-11, its role in respiratory infections caused by major veterinary pathogens, and its potential applications in disease intervention. By critically evaluating the current evidence and identifying knowledge gaps, we aim to provide a theoretical framework to guide future research in this emerging field.

## 2 Activation mechanism and signaling pathway of the Caspase-11 inflammasome

### 2.1 Molecular characteristics and expression regulation of Caspase-11

Kayagaki et al. first identified Caspase-11, a member of the Caspase family, as a critical component of the non-canonical inflammasome (10). It is expressed primarily in macrophages, neutrophils, epithelial cells, and other immune or parenchymal cell types, the human homologs of which are Caspase-4 and Caspase-5 (21). In mammals such as pigs and cattle, functional homologs of Caspase-11 have been identified. These encoded proteins possess a conserved CARD domain and protease active site, indicating that the molecule is highly conserved in both evolution and function (22, 23). Caspase-11 expression can be induced by cytokines such as IFN-γ and TNF-α. Specifically, IFN-γ promotes its transcription via the STAT1 signaling pathway (24). NF-κB signaling also plays a critical role in its transcriptional regulation. Additionally, LPS significantly upregulates Caspase-11 expression via the TLR4-NF-κB pathway (25–28).

### 2.2 Fine-tuning of Caspase-11 activation

In addition to the activation triggered by direct LPS binding described above, the activation efficiency of Caspase-11 is also finely regulated by various cofactors. Guanylate-binding proteins (GBPs) can disrupt bacterial vesicles or the bacterial outer membrane, promoting the release of LPS into the cytoplasm (29). High-mobility group box 1 (HMGB1), which acts as an LPS chaperone, enhances the ability of LPS to enter the cytoplasm (30). In terms of negative regulation, the cAMP-PKA signaling axis can inhibit Caspase-11 activity by phosphorylation (31). Additionally, when present at low concentrations, oxidized phospholipids can competitively bind to the CARD domain of Caspase-11, thereby blocking its activation (32). Together, these positive regulatory mechanisms (GBPs, HMGB1) and negative regulatory mechanisms (PKA-mediated phosphorylation, low-concentration oxidized phospholipids) ensure that Caspase-11 is sufficiently activated to defend against infection while being restrained from over activation that would otherwise cause immunopathological damage. Notably, pathogens have also evolved various escape mechanisms, such as modifying the acetylation patterns of LPS or secreting effector proteins that directly inhibit Caspase-11 activity (33–39).

### 2.3 Activation pattern of the Caspase-11 inflammasome

Activation of the Caspase-11 inflammasome depends on direct recognition of cytosolic LPS and can be divided into two stages. The first is the priming stage: upon pathogen invasion, host cells recognize pathogen-associated molecular patterns (PAMPs) via TLR4 or other pattern recognition receptors, activating the NF-κB signaling pathway and promoting the expression of the Caspase-11 precursor as well as pro-IL-1β and pro-IL-18 (10). The second stage is the activation stage. LPS is released into the cytoplasm via bacterial outer membrane vesicles (OMVs) or pathogen lysis (40). It then directly binds to Caspase-11, inducing its oligomerization and conformational changes (22). This activates the protease activity of Caspase-11 (41). Although this process is often accompanied by intermolecular autoprocessing, this cleavage event is not required for its ability to cleave GSDMD (42).

**Figure 1:**
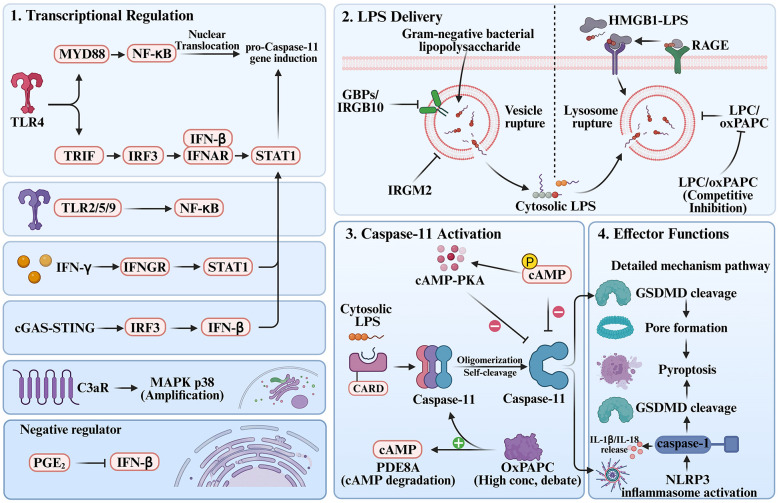
Schematic illustration of the non-canonical Caspase-11 inflammasome activation pathway. The activation process of the non-canonical Caspase-11 inflammasome is divided into four stages: (1) Transcriptional regulation of pro-Caspase-11 via TLR4-NF-κB, TLR4-TRIF-IFN-β-STAT1, IFN-γ-STAT1, and other pathways; (2) LPS delivery into the cytosol, which is facilitated by GBPs/IRGB10-mediated vesicle rupture and negatively regulated by LPC/oxPAPC; (3) Caspase-11 activation upon cytosolic LPS binding, leading to oligomerization and self-cleavage; (4) Effector functions, including GSDMD cleavage to induce pyroptosis and NLRP3 inflammasome activation to promote IL-1β/IL-18 maturation. Negative regulators such as cAMP-PKA and positive modulators are also indicated.

Activated Caspase-11 functions in two ways. First, it directly cleaves GSDMD. This leads to the formation of membrane pores and triggers pyroptosis. Consequently, proinflammatory factors and damage-associated molecular patterns (DAMPs) are released (9). Second, it activates the NLRP3 inflammasome, promoting Caspase-1 activation and the maturation of IL-1β and IL-18, thereby amplifying inflammatory signals (43, 44). Moretti et al. further demonstrated that Caspase-11 directly binds to the NACHT domain of NLRP3, thereby promoting its oligomerization and markedly enhancing its activation efficiency (43). In addition, Caspase-11 can influence host clearance of invasive pathogens by modulating macrophage phagocytosis and promoting neutrophil recruitment to infection sites (45–49). The activation and regulatory mechanisms are illustrated in Figure 1.

### 2.4 Functional conservation and species specificity of Caspase-11 homologous molecules in livestock

The functional homolog of Caspase-11, Caspase-4, has been identified in mammals such as pigs and cattle. Although the CARD domains and protease active sites of various homologous molecules are highly conserved, species differences exist in their regulatory elements and expression patterns (21, 23). Sequence and structural differences in Caspase-11 homologs across species may influence their recognition and activation by LPS, thereby contributing to functional specificity (21, 50).

For a comprehensive understanding of the evolutionary history of Caspase-4/5/11, readers are directed to a recent review (51). Furthermore, besides homologs of human and murine caspases, mammals such as pigs and cattle possess additional inflammatory caspases including caspase-15 and caspase-16, which may serve redundant or species-specific functions (52, 53).

**Figure 2:**
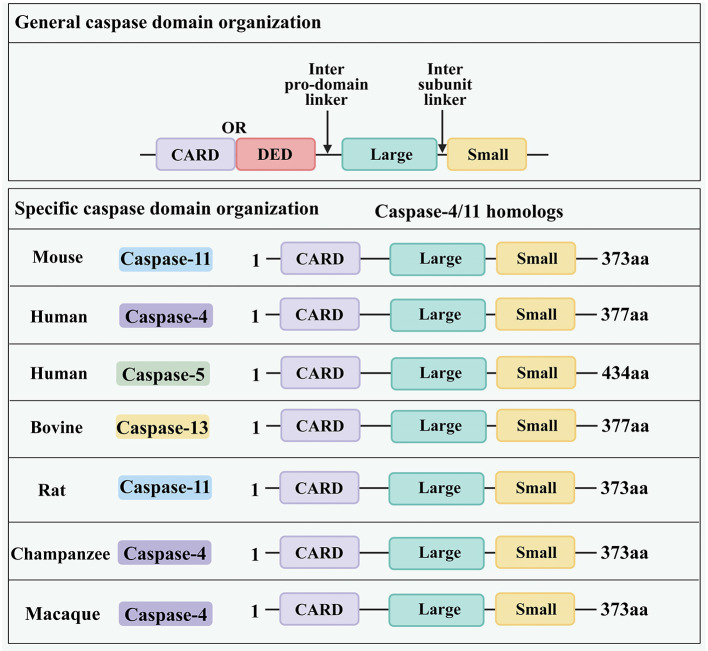
Domain organization of Caspase-4/11 homologs in different species. Schematic representation of the domain structures of Caspase homologs derived from different species, including murine (mouse, rat), primate (human, chimpanzee, macaque), and bovine. All family members contain an N-terminal Caspase recruitment domain (CARD), followed by a large subunit (p20) and a small subunit (p10). Bovine Caspase-13 is the functional ortholog of human Caspase-4 and murine Caspase-11. *Caspase-13 is the bovine ortholog caspase-4 and does not exist in mouse or human. *CARD, caspase recruitment domain; Large, large subunit (P20); Small, small subunit (P10). Domain order is from N-terminus to C-terminus.

The structural conservation and species-specific differences among Caspase-11 homologs are illustrated in Figure 2, which compares the domain organizations of representative homologs from rodents, primates, and veterinary species.

## 3 Role of the Caspase-11 in respiratory tract infections in veterinary species

### 3.1 Bacterial respiratory pathogens

#### 3.1.1 Gram-negative bacteria

Caspase-11 is essential for gram-negative veterinary respiratory bacterial infections (19) especially in *Pasteurella multocida*. *P. multocida* is an important pathogen responsible for swine atrophic rhinitis and bovine pneumonia, with documented roles in host immune modulation (54) and with infection activating Caspase-11 expression in respiratory cells (55). In a mouse model, Yang et al. reported that *P. multocida* delivers LPS to the cytoplasm through the secretion of outer membrane vesicles. This activates the Caspase-11-GSDMD pathway, leading to alveolar macrophage pyroptosis and the release of proinflammatory factors such as IL-1β and TNF-α. These events subsequently trigger inflammatory infiltration and damage to lung tissue. Caspase-11 knockout significantly reduced the degree of inflammation and mortality following *P. multocida* infection (56). Tang et al. reported that Caspase-11 mediates lethal inflammatory responses in a sepsis model of gram-negative bacterial infection (55). Notably, current studies on *P. multocida* and Caspase-11 are based on mouse gene knockout models. The function of homologous Caspase-11 molecules has not yet been validated in natural hosts such as pigs and cattle. Considering that LPS recognition sensitivity may differ between murine Caspase-11 and Caspase-4 in livestock and that the promoter region of Caspase-4 in these species contains species-specific regulatory elements, whether existing conclusions can be directly extrapolated to veterinary clinical settings remains to be experimentally confirmed.

*Klebsiella pneumoniae* is an important zoonotic Gram-negative pathogen (57). Caspase-11 plays a dual role in *Klebsiella pneumoniae* infection, and this role is context-dependent. In local pulmonary infection, Caspase-11 contributes to host defense by imprisoning bacteria and limiting their spread through local coagulation (58). In systemic infection, Caspase-11 and NLRP3 synergistically damage the mitochondrial function of macrophages, reduce their bactericidal ability, and exacerbate infection and lung injury (59). Notably, Current studies on the dual functions of Caspase-11 in *Klebsiella pneumoniae* infections are based on mouse knockout models (58, 59); the functions of Caspase-11 homologous have not yet been validated in natural veterinary hosts such as pigs, cattle, and horses. Given that mouse Caspase-11 and Caspase-4 in livestock may differ in their sensitivity to LPS recognition (60), and that the promoter regions of Caspase-4 in these species contain species-specific regulatory elements, further experimental validation using natural host models is required to determine whether Caspase-11 plays a functional role in *Klebsiella pneumoniae* infections in the veterinary field and whether it exhibits a similar “local defense-systemic damage” dual effect.

Beyond *P. multocida* and *Klebsiella pneumoniae*, emerging evidence implicates Caspase-11 in host defense against other Gram-negative pathogens of veterinary relevance. *Acinetobacter baumannii*, an opportunistic pathogen increasingly recognized in veterinary settings and associated with pneumonia outbreaks in animals, has been shown to activate the Caspase-11 non-canonical inflammasome. Using a murine model of pulmonary infection, Wang et al. demonstrated that Caspase-11 deficiency impaired bacterial clearance from the lungs and systemic organs, exacerbated pulmonary pathological changes characterized by extensive inflammatory infiltration and consolidation, and significantly reduced host survival. These findings indicate that Caspase-11 exerts a protective role in host defense against A. baumannii pulmonary infection, highlighting the broad recognition capacity of this inflammasome for diverse Gram-negative pathogens (61). Similarly, *Escherichia coli*, a common cause of respiratory infections and septicemia in livestock (62), activates the Caspase-11 pathway through outer membrane vesicles (OMVs) that deliver LPS into the host cytosol via endocytosis (40). This OMV-mediated mechanism provides a critical mechanistic link between extracellular *E. coli* infection and cytosolic LPS sensing, underscoring the relevance of the non-canonical inflammasome in host defense against this pathogen. Collectively, these findings underscore that the functional relevance of Caspase-11 extends to a wider spectrum of Gram-negative species than currently appreciated. However, as with *P. multocida* and *Klebsiella pneumoniae*, these observations are predominantly derived from murine models, and functional validation in natural veterinary hosts such as pigs, cattle, and goats remains a critical priority for future research.

#### 3.1.2 Gram-positive bacteria

Compared with that of gram-negative bacteria, the role of Caspase-11 in Gram-positive respiratory infections of livestock remain poorly characterized. Nevertheless, accumulating evidence suggests that Caspase-11 contributes to host immune responses against certain Gram-positive pathogens through indirect mechanisms distinct from direct lipopolysaccharide (LPS) sensing (as reviewed in (63)).

*Staphylococcus aureus* is a common opportunistic pathogen in livestock, and is capable of causing pneumonia, mastitis, and other infections in pigs and cattle (1, 4). Beyond Gram-negative bacterial infections, Caspase-11 has also been implicated in host responses against Gram-positive pathogens. Using a murine model, Krause et al. demonstrated that Caspase-11 facilitates the intracellular survival of methicillin-resistant *S. aureus* (MRSA) by preventing the recruitment of mitochondria to bacteria-containing vacuoles, thereby limiting mitochondrial reactive oxygen species (ROS)-mediated bacterial clearance (64). Similarly, Hara et al. showed that during Listeria monocytogenes infection, NLRP6 recognizes lipoteichoic acid and recruits Caspase-11 to promote IL-18 production, exacerbating systemic infection (65). These findings collectively indicate that the functional relevance of Caspase-11 extends beyond Gram-negative bacterial infections to include host responses against Gram-positive pathogens.

*Streptococcus suis* represents one of the most significant Gram-positive pathogens in the swine industry, causing septicemia, meningitis, and pneumonia (4, 5, 8). In addition to being isolated from swine, *S. suis* has been isolated from clinical cases of bronchopneumonia in cattle, underscoring its relevance across livestock species (66). Mechanistic studies have revealed that *S. suis* serotype 2 activates the NLRP3 inflammasome via the pore-forming toxin suilysin (SLY), and Caspase-1/11 deficiency substantially reduces interleukin (IL)-1β release and increases survival in infected mice (67). More recently, Shi et al. reported that membrane vesicles derived from *S. suis* serotype 2 induce pyroptosis in endothelial cells through the NLRP3/Caspase-1/GSDMD canonical inflammasome pathway. Notably, these vesicles did not activate the Caspase-4/5 pathway, which is a human homolog of murine Caspase-11, suggesting that as a gram-positive bacterium lacking LPS, *S. suis* does not directly trigger non-canonical inflammasome activation. Thus, whether *S. suis* can indirectly regulate Caspase-11 activity through cross-talk with other inflammatory pathways remains to be investigated (68).

Collectively, while direct activation of Caspase-11 by Gram-positive pathogens appears to occur through mechanisms distinct from those associated with canonical LPS sensing, a growing body of evidence supports the functional relevance of Caspase-11 in modulating inflammasome responses (63, 65, 67). *S. aureus* and *S. suis* are established respiratory pathogens in livestock, and ongoing research continues to elucidate the complex interactions between these bacteria and host inflammatory pathways. However, functional validation of the involvement of Caspase-11 in natural livestock hosts remains limited and represents a critical direction for future investigations.

### 3.2 Viral respiratory pathogens

Currently, the role of Caspase-11 in viral infections has attracted widespread attention. Previous studies have indicated that Caspase-11 exerts a bidirectional regulatory function during viral infection. In animal-restricted viral models, Caspase-11 can inhibit viral replication by regulating cellular metabolism, thereby exerting an antiviral protective effect (69). In zoonotic viral infections, influenza virus induces Caspase-11-mediated aggravation of lung injury in low-humidity environments (70). Rabies virus infection reveals that Caspase-1/11 deficiency only exacerbates symptoms of attenuated strains but has no significant effect on virulent strains (71). Leishmania RNA virus inhibits Caspase-11 activation via TLR3 and autophagy-related pathways to promote its own replication (72). In human-restricted viral infections, Coxsackievirus B3 promotes Caspase-11-mediated pyroptosis and myocardial damage (73). With respect to research on animal coronaviruses, the current understanding remains very limited and relies primarily on the SARS-CoV-2 model, a human-emerged coronavirus. Eltobgy et al. reported that Caspase-11 primarily exacerbates immunopathological damage and promotes inflammatory responses and immune thrombosis without affecting viral replication; moreover, Caspase-11 knockout significantly alleviates lung injury and inflammatory responses. Rodrigues et al. further confirmed that its human homolog, Caspase-4, similarly promotes NLRP3 activation and accelerates disease progression (74, 75). Whether Caspase-11 plays similar roles in veterinary animal coronavirus infections remains to be explored.

In the context of viral infections relevant to veterinary species, Li et al. recently demonstrated that African Swine Fever virus (ASFV) infection regulates pyroptosis by cleaving gasdermin A via active caspase-3 and caspase-4 in porcine macrophages, highlighting a novel viral evasion mechanism targeting the non-canonical pathway (76).

However, significant gaps remain in the current research. First, most of the aforementioned studies have focused on mouse models and human-associated viruses. In the field of veterinary medicine, research on viral diseases in economically important animals (such as pigs, cattle, sheep) is still in its infancy; the expression patterns and activation mechanisms of Caspase-11 in animal primary cells and target organs, as well as its dynamic relationship with pathogen replication, remain unclear. Second, respiratory diseases in livestock often manifest clinically as mixed infections, including bacterial-viral coinfections, mixed bacterial infections, or multiple viral coinfections. However, existing studies on Caspase-11 have focused primarily on single pathogens; thus, the activation mechanisms, regulatory networks, and balancing role of Caspase-11 between immune protection and immunopathology under conditions of mixed infection remain unexplored. Furthermore, none of studies have explored the cross-regulatory mechanisms between Caspase-11 and host-specific immune pathways in animals. The regulatory effects of intervention strategies targeting Caspase-11 in animal pathogen infections, as well as their clinical application potential, also urgently require evaluation.

## 4 Regulatory mechanism and targeted application potential of the Caspase-11

### 4.1 Regulatory effects of natural regulatory molecules on Caspase-11

The activation of the Caspase-11 inflammasome is finely regulated by a variety of natural molecules. These regulatory molecules inhibit or increase the activity of Caspase-11 through different mechanisms to maintain the balance of the inflammatory response (23).

HMGB1 can act as an LPS delivery protein to increase activation (30), whereas guanylate-binding proteins (GBPs) promote the release of bacterial LPS into the cytoplasm, further increasing its activation (77). In human and mouse models, silymarin, quercetin, and morin have been shown to inhibit Caspase-11 self-cleavage and GSDMD activation, thereby blocking pyroptosis (78–80). Wedelolactone downregulates Caspase-11 expression at the transcriptional level by inhibiting the NF-κB pathway (81). These mechanistic breakthroughs provide an important theoretical foundation and research direction for the development of novel anti-inflammatory agents targeting Caspase-11 in livestock.

### 4.2 Caspase-11-targeted prevention and control strategy development

#### 4.2.1 Anti-inflammatory drug design

The molecular mechanisms governing Caspase-11 inflammasome activation offer multiple nodes for therapeutic intervention. The selective Caspase-1 inhibitor VX-765 (Belnacasan) is widely utilized as a pharmacological tool to distinguish between classical and non-canonical inflammasome pathways. Given that VX-765 does not inhibit Caspase-11 activity, the blockade of cell death following treatment is generally interpreted as indicating classical NLRP3-Caspase-1 pathway activity. However, this interpretation warrants caution, as non-canonical Caspase-11 activation can engage the NLRP3-Caspase-1 axis downstream, potentially rendering the pathway partially susceptible to VX-765 inhibition under certain experimental conditions (82). Consequently, definitive pathway assignment necessitates complementary validation strategies, including Caspase-11 genetic ablation, assessment of GSDMD cleavage, or the application of specific Caspase-11 inhibitors.

In addition to upstream inflammasome components, direct targeting of the pyroptotic executioner GSDMD represents an alternative therapeutic strategy. Necrosulfonamide, which blocks the pore-forming activity of GSDMD, has been shown to effectively inhibit pyroptosis and mitigate inflammatory tissue damage (83). In a separate approach, Wang et al. demonstrated that glycyrrhizin attenuates Caspase-11 activation by competitively inhibiting the binding of HMGB1 to LPS, thereby reducing inflammatory responses in a murine model of LPS-induced acute lung injury (84).

Importantly, the translational potential of these inhibitors in livestock species remains largely unexplored. Species-specific variations in drug metabolism, pharmacokinetic profiles, and target protein binding affinities may significantly influence therapeutic efficacy and safety outcomes. Future studies should prioritize the evaluation of these inhibitors in clinically relevant large animal models to facilitate their potential application in veterinary medicine.

#### 4.2.2 Vaccine adjuvant optimization

The ability of Caspase-11 inflammasome activation to enhance immune responses makes its regulatory mechanisms a promising target for vaccine adjuvant optimization. For example, endogenous Caspase-11 ligands can mediate the release of IL-1 release from living dendritic cells and increase the intensity of the immune response (85). This provides a new avenue for the development of low-toxicity, high-efficiency vaccine adjuvants. In addition, the activation of Caspase-11 by LPS and its low-toxicity derivatives also suggests its potential application as a vaccine adjuvant (10, 85).

#### 4.2.3 Application of gene editing technology

The application of CRISPR-Cas9 and other gene-editing tools to precisely engineer Caspase-11 homologs in livestock represents a promising strategy for developing disease-resistant breeds with optimized inflammatory response regulation. Although excessive Caspase-11 activation aids in the clearance of certain gram-negative bacteria, it simultaneously represents a critical contributor to severe inflammatory pathology. Therefore, moderate inhibition of its activity through gene editing represents a potential strategy to balance immune protection and immunopathological damage. In this context, MT3 has been shown to inhibit Caspase-11 activation through zinc ion regulation (86). Additionally, Eren et al. demonstrated that Irgm2 and Gate-16 act synergistically to suppress noncanonical Caspase-11 inflammasome activation triggered by gram-negative bacteria (87). These studies on endogenous inhibition mechanisms provide potential molecular targets for regulating Caspase-11 activity through gene editing and for exploring the cultivation of disease-resistant livestock breeds. However, current studies on these targeting strategies are based on mouse models, and their conservation and feasibility in livestock remain to be further verified (86, 87).

## 5 Research prospects and challenges

Although some progress has been made in understanding the role of the Caspase-11 inflammasome in veterinary respiratory pathogen infections, significant challenges remain. First, functional validation of Caspase-11 homologous molecules in livestock remains insufficient. Their activation mechanisms and regulatory networks vary across different species and cell types, necessitating further *in vitro* and *in vivo* studies. Second, veterinary respiratory diseases often involve mixed infections, making the role of Caspase-11 in multipathogen co-infection considerably more complex. The establishment of a mixed infection model that more closely reflects clinical conditions represents an important direction for future research. In addition, the specificity and safety of Caspase-11 inhibitors and activators still require optimization to ensure their efficacy and to minimize residual risks in livestock.

Future research can be pursued in depth across several interrelated levels. At the molecular structural level, crystallographic analysis of Caspase-11 homologs in livestock will provide direct insights into their interaction mechanisms with key molecules such as LPS and GSDMD. At the infection model level, given that clinical cases frequently involve mixed infections, more realistic multipathogen coinfection models need to be established. These findings help elucidate the cross-talk and synergistic regulatory networks between Caspase-11 and other inflammasomes (43, 44). At the translational application level, the development of novel anti-inflammatory drugs and vaccine adjuvants targeting Caspase-11 holds promise for providing new strategies for the prevention and control of veterinary respiratory diseases (88). In addition, the application of multiomics techniques, including transcriptomics, proteomics, and metabolomics will help systematically delineate the molecular landscape of Caspase-11 activation and facilitate the discovery of novel intervention targets.

## 6 Conclusion

In summary, the Caspase-11-mediated non-canonical inflammasome has emerged as a key regulator of anti-infective immunity in the respiratory tract of livestock. By directly recognizing cytoplasmic lipopolysaccharides, this inflammasome mediates pyroptosis and amplifies inflammatory signals, serving an indispensable role in defending against gram-negative bacterial invasion and preserving host immune homeostasis. Existing evidence has demonstrated that Caspase-11 activation is strongly dependent on context and functional duality in response to common veterinary respiratory pathogens, including *P. multocida* and *K. pneumoniae*: moderate activation facilitates pathogen clearance, whereas excessive activation precipitates uncontrolled inflammation, exacerbates lung tissue damage, and triggers immune dysregulation that may culminate in systemic pathology. Notably, Caspase-11 also contributes to inflammatory regulation through indirect pathways in the context of gram-positive bacterial and viral infections, indicating its broader pathophysiological relevance in respiratory polymicrobial infections and complex diseases.

Although some progress has been made in recent years, the structural basis, signaling networks, and species-specific mechanisms of Caspase-11 homologs in livestock remain poorly understood. Caspase-11-targeted anti-inflammatory agents, vaccine adjuvants, and gene-edited breeding for disease resistance are still at an early stage of development. Looking ahead, the rapid advancement of structural biology, gene editing, and multiomics technologies are poised to propel several promising directions: elucidating the precise mechanisms of Caspase-11 in respiratory pathogen infections in livestock; establishing mixed infection models that more faithfully recapitulate clinical scenarios; and developing targeted interventions with high specificity and favorable pharmacokinetic profiles.

Collectively, these findings reveal that Caspase-11 is both a cornerstone for deciphering inflammatory mechanisms in veterinary respiratory diseases and a compelling target for precision-based strategies in epidemic disease control. Systematic elucidation of its function and regulatory network would provide critical theoretical foundations for developing novel anti-inflammatory therapeutics, efficient vaccine adjuvants, and disease-resistant livestock breeds, holding significant scientific value and translational promise for improving respiratory disease control in livestock as well as advancing the sustainable development of animal husbandry.
